# Acceptability of Mobile Phone Technology for Medication Adherence Interventions among HIV-Positive Patients at an Urban Clinic

**DOI:** 10.1155/2013/670525

**Published:** 2013-08-13

**Authors:** Christopher W. T. Miller, Seth Himelhoch

**Affiliations:** Department of Psychiatry, University of Maryland School of Medicine, 737 West Lombard Street, Room 516, Baltimore, MD 21201, USA

## Abstract

Mobile phone technology is increasingly used to overcome traditional barriers limiting access to care. The goal of this study was to evaluate access and willingness to use smart and mobile phone technology for promoting adherence among people attending an urban HIV clinic. One hundred consecutive HIV-positive patients attending an urban HIV outpatient clinic were surveyed. The questionnaire evaluated access to and utilization of mobile phones and willingness to use them to enhance adherence to HIV medication. The survey also included the CASE adherence index as a measure of adherence. The average age was 46.4 (SD = 9.2). The majority of participants were males (63%), black (93%), and Hispanic (11.4%) and reported earning less than $10,000 per year (67.3%). Most identified themselves as being current smokers (57%). The vast majority reported currently taking HAART (83.5%). Approximately half of the participants reported some difficulty with adherence (CASE < 10). Ninety-six percent reported owning a mobile phone. Among owners of mobile phones 47.4% reported currently owning more than one device. Over a quarter reported owning a smartphone. About 60% used their phones for texting and 1/3 used their phone to search the Internet. Nearly 70% reported that they would use a mobile device to help with HIV adherence. Those who reported being very likely or likely to use a mobile device to improve adherence were significantly more likely to use their phone daily (*P* = 0.03) and use their phone for text messages (*P* = 0.002). The vast majority of patients in an urban HIV clinic own mobile phones and would use them to enhance adherence interventions to HIV medication.

## 1. Introduction

Optimal adherence to HAART strongly predicts HIV viral suppression [1] and is linked to improved survival [2]. Low adherence to HAART in the United States is common with approximately only 50%–70% of prescribed doses taken [3, 4]. One meta-analysis found that those who participated in HAART-related adherence-enhancing interventions were significantly more likely to achieve 95% adherence and viral load suppression compared to the control condition [5]. A recent systematic review analyzed findings from 31 projects assessing use of SMS (short message service) technology, most for patients with HIV/AIDS in developing countries [6]; the findings pointed towards SMS as a promising and mostly well-accepted intervention strategy for use in healthcare.

The utilization of technology-based health applications for disease prevention and management has been progressively explored and expanded upon [7–11]. Promising results have been found for a number of conditions (e.g., diabetes mellitus, asthma, nicotine use, and obesity) with use of mobile phone technology [12, 13] as well as more specifically with text messaging [14, 15]. This extends to people living with HIV (PLWH), and the utilization of technology in improving education, adherence, and biological markers (CD4 count and viral load) has shown promising results [16–18]. As mobile technology access has become increasingly widespread, this seems to be an ideal tool to reach a large segment of PLWH in a cost-effective manner [19]. Thus far, technologies employed with this population have included computer-based programs [20, 21], mobile devices (both for phone counseling sessions [22–24] as well as text messaging) [16–18, 25, 26], and paging devices [27]. These have been used as educational resources and as reminders for taking HAART, in an effort to improve disease control. 

As mobile technology evolves, many people are updating their devices to smartphones, which offer a greater number of features and may allow for more creative and interactive interventions for improving adherence in PLWH. Ability and willingness to utilize this technology are key factors in ensuring the success of planned intervention strategies. This study aimed to evaluate access and willingness to use smart and mobile phone technology for promoting adherence among people attending an urban HIV clinic. To our knowledge, this is the first study to specifically address potential incorporation of smartphones in treatment paradigms.

## 2. Materials and Methods

### 2.1. Study Design and Sample

This single site study sought to evaluate access and willingness to use smart and mobile phone technology for promoting adherence among people attending an urban HIV clinic. Eligibility for the study included having a diagnosis of HIV and attending the clinic on the day of recruitment. Potential participants were recruited in May 2012. One hundred consecutive adult (18 years of age and older) HIV-positive patients attending an urban HIV outpatient clinic were surveyed. All eligible participants on the day of recruitment were given the opportunity to participate in the study. None declined. Everyone enrolled in the study completed the survey. The study was reviewed by the University of Maryland Baltimore's Institutional Review Board and given an exempt status. Participants were paid $10 for completing the study survey. 

### 2.2. Assessment Instruments

The study questionnaire evaluated access to and utilization of mobile phones and willingness to use them to enhance adherence to HIV medication. The present study was given an exempt status by the Institutional Review Board (IRB). All patient data was de-identified, and confidentiality was thus preserved. All study procedures occurred behind a locked, closed door.

The questionnaire included the following sections.

*Demographic Questionnaire*. Standardized questions were used to gather demographic information including age, gender, race, education, job description, employment history, and smoking status.

*Ownership, Access, and Willingness to Use Mobile or Smartphone Devices.* Questions were created de novo or adapted from that questions that were developed by the Pew Research Center, Pew Internet and American Life Project, to evaluate use of mobile and smartphone devices. Questions regarding ownership (including number of phones, types of phones, and service plan utilized); access (e.g., internet); utilization (including utilization of text messaging, phone calling, software applications, and social network sites), and willingness to use mobile devices to access and enhance medical care were employed.

The Pew questionnaire assessed the following categories of cell phone use: (1) take a picture; (2) send or receive text messages; (3) access the internet; (4) send or receive email; (5) record Video; (6) download applications; (7) look for health or medical information online; and (8) check bank account balance or do any online banking. These categories were stratified in the Pew questionnaire according to the following demographic data: gender, age, race/ethnicity, annual household income, and education level. The Results section will outline the modifications to the questionnaire that were utilized in the present study.

*Center for Adherence Support Evaluation (CASE) Adherence Index [28].* The CASE index is a 3-item adherence questionnaire. The three self-reported measures of adherence are as follows: A1—frequency of “difficulty taking HIV medications on time” (no more than two hours before or two hours after the time the patient's doctor instructed to take the medication); A2—“average number of days per week at least one dose of HIV medications was missed”; and A3—“last time missed at least one dose of HIV medications.” With regards to scoring, A1 contributes a possible range of one to four points, while A2 and A3 each contributes one to six points. A score of greater than 10 is associated with good adherence. A score of 10 or less is associated with poor adherence. For those who reported missing a dose of HAART medication on the CASE adherence index were asked questions about the reasons for which they missed taking medications from the AACTG Adherence Instrument [29]. The AACTG Adherence Instrument includes 11 reasons a person may not have taken HIV medication. Questions are rated on a 4-point likert scale (never, rarely, sometimes, and often). 

### 2.3. Analysis

Univariate distributions included percentages for dichotomous variables and means for normally distributed continuous variables. Comparison of means was made using two-sided *t*-tests, while comparison of percentages was made using the chi-square of Fischers exact method. Data was analyzed using SAS. All reported *P* values are two-sided. 

## 3. Results

### 3.1. Participant Characteristics

The average age was 46.4 years (SD = 9.2). The majority of participants were males (62.6%), black (93.0%), and Hispanic (11.4% (*n* = 9)) and reported earning less than $10,000 per year (67.3%). Most identified themselves as being current smokers (57%). The vast majority reported currently taking HAART (83.5%) (see Table 1).

### 3.2. Ownership and Utilization of Mobile Phones

Ninety-six percent (*n* = 100) reported owning a mobile phone. Among owners of mobile phones 47.4% reported currently owning more than one device, 75% reported using it for one year or more, and 81% report using it 5–7 days a week. Forty-two percent reported having a phone plan without a contract and the vast majority report never having to discontinue service because they could not afford it (66%). Nearly all reported using their cell phone to make telephone calls (92%). Fifty-nine percent used their phones for texting. Those who report using text messaging were significantly more likely to be younger (42.1 years ±9.8 versus 51.2 years ±7.3) compared to those who did not report texting. No other demographic factor was significant in bivariate analysis. Thirty-four percent used their phone to search the Internet. Nearly half used their phone to set alarms (47%); schedule events using a calendar (48%); and use the clock (49%) (see Table 2).

Over a quarter (28.7%) reported owning a smartphone. Those who reported owning a smartphone were significantly more likely to be younger (39.8 years ±8.6 versus 47.3 ± 11.6, *t* = 3.20, *P* = 0.002) and more likely to have incomes greater than $10,000 per year compared to those with higher incomes (76.9% versus 40.7%, *P* = 0.03). No other demographic factor was significant in bivariate analysis.

### 3.3. Adherence to HAART

Among those reporting taking HAART, approximately half (51% (*n* = 42)) had poor adherence as evaluated by the Center for Adherence Support Evaluation (CASE) adherence index (i.e., CASE < 10). Bivariate analysis did not demonstrate any significant differences in demographic characteristics comparing those with poor adherence (i.e., CASE < 10) to those with good adherence (i.e., CASE > 10). Among those reporting ever missing a dose of HIV medication (*n* = 25) the most frequent reasons included (1) simply forgetting (52% reported “sometimes”); (2) sleeping through the dosage (52% reported “sometimes”); (3) being away from home (39% reported “sometimes” and 9% reported “often”); and (4) having a change in daily routine (40% reported “sometimes” and 4% reported “often”) (see Table 3).

All participants were asked how likely they would be to use a cell phone that could remind them every day when to take HIV medication. The vast majority reported (69%) that they were “likely” or “very likely” to use it. Those who said they were “very likely” or “likely” to use a cell phone to remind them to take medication were significantly more likely to report ever using their phone to receive or send text messages (68.3% versus 33.3%, *χ*2 = 9.97, *P* < 0.002); more likely to use the phone 7 days a week (71.9% versus 48.1%, fisher exact test, *P* < 0.03); and use their phone to set alarms (53.9% versus 29.7%, *χ*2 = 3.47, *P* < 0.04). 

## 4. Discussion

The vast majority of patients in an urban HIV clinic own mobile phones and would use them to enhance adherence interventions to HIV medication. In particular we found that nearly all (96%) of the participants surveyed owned a mobile phone and nearly half (47.4%) reported currently owning more than one device. Sending or receiving text messaging was the most common additional feature that was used on the mobile devices. Although 58% reported sending or receiving a text message, this was somewhat less than the 80% of cell phone owners who are reported nationally to use their cell phone to send or receive a text message [30]. Potential reasons for this discrepancy is that the population studied is on average of an older age and thus may be more used to using landline phones. In addition, the overall education level of the population is low, and this may impact comfort level with regards to typing messages.

Nearly 70% reported that they would use a mobile device to help with HIV adherence. Those who reported being very likely or likely to use a mobile device to improve adherence were significantly more likely to use their phone daily (*P* = 0.03) and use their phone for text messages (*P* = 0.002). Text messaging has been shown to help with multiple aspects of care in a number of medical conditions (including HIV), with demonstrable improvement in medication adherence and biological markers [18, 31]. A number of studies have addressed how best to employ this instrument in optimizing compliance [26, 32, 33]. Promising results using text messaging have been found in several studies [16–18, 25, 26, 33]. One of the seminal studies in this area was a multisite randomized controlled trial (RCT) utilizing mobile device technology to improve adherence to highly active antiretroviral therapy (HAART)—the WelTel Kenya1 study [33]—in which a single SMS was sent to the participants at the beginning of the week asking, “How are you?” compared to a control group receiving standard care. In terms of adherence, 62% of the intervention groups participants had greater than 95% compliance, compared to 50% compliance in the control group. The intervention group also reported greater viral suppression at 12 months. Another RCT in Kenya reported a statistically significant improvement in HAART adherence at 48 weeks in the group receiving weekly SMS reminders, as compared to the control group [26]. Interestingly, the latter study did not find a significant increase in adherence in the group receiving daily messages. 

Individual patient characteristics need to be recognized in order to maximize intervention responses, particularly with regards to patterns of cell phone use. Individuals are more or less comfortable with certain aspects of their cell phones, and this degree of comfort will influence how likely they are to participate in proposed interventions. Sidney et al. conducted a study in which 139 adult HIV patients who were on regular antiretroviral therapy received a weekly interactive call as well as a neutral pictorial SMS [34]. The participants were requested to report what their adherence had been like the previous day; in the case of failure to respond, additional calls to the individual patients were made. In their study, only 11% of patients preferred only receiving an SMS, while 87% indicated a preference for the phone call reminder. 

It should be noted that participants seemed to have a greater knowledge of the phone-related functions as compared with the SMS (e.g., calendar, alarm, and listening to music). This correlates with the findings in our study and makes logical sense; participants who expressed being more likely to use mobile phone reminders were those who use a greater number of functions on their phones, and use them on a more regular basis. How interactive an intervention is may also be a key component, as the individual may variably feel more or less involved in their own care, depending on the investment the intervention team makes in ensuring they are reading the messages, as opposed to being just a passive recipient of an impersonal reminder. In an RCT performed at a teaching hospital in Boston, 23 HIV-positive participants were randomized to either receive a mobile phone message with content selected by the patient from a list of options (e.g., weather report, news, and Bible verses) or to receive a beeper [17]. Participants using the mobile device were encouraged to respond to the reminder (devices would continue to beep until this occurred), whereas the pager would only beep once, irrespective of the participant's response. When using mixed measures of adherence, there was a statistically significant difference between the two groups at both three (*P* = 0.0129) and six weeks (*P* = 0.002), favoring the mobile phone group. Results may have been favored by the fact that there were constant reminders until a response was obtained in the intervention groups; this is important due to possible user fatigue over time—there was a decrease in participants' responses to text messages between weeks three and six of the study. To exemplify further, in the study by Simoni et al., there was no improvement at any point of the intervention in terms of antiretroviral adherence when utilizing a two-way pager system (though there was improvement in biological markers of illness) [27]. One of the potential strategies to assist individuals who do not utilize text messaging would be to offer training in the use of a self-contained mobile application.

Randomized controlled trials (RCTs) which intervened sending individuals text messages on a weekly basis reported significant improvement in adherence [18, 26]. In the study by da Costa et al. with Brazilian women with HIV/AIDS [25], there was a nonsignificant improvement in medication compliance in the group receiving SMS multiple times during the week, and over 63% of participants reported that the intervention helped them to take their medications more regularly. Intermittently sending reminders (as opposed to everyday) has been utilized as a way of keeping patients from underestimating the importance of the messages, as well as focusing on days which have been shown to be more strongly associated with noncompliance (e.g., weekends) [25, 35]. User fatigue and trivializing of frequent messaging may be hampering factors in maintaining compliance. 

In our study, approximately a quarter reported owning a smartphone, which was considerably less than the national smartphone ownership of 52% reported by the Pew Internet and American Life Project [30]. This difference may be due to the cost associated with owning a smartphone, the additional cost of the data-plan, and possibility of a less robust market penetration of these devices at the time of the study. However, with the increased penetration of smartphone ownership only growing, there is a greater opportunity to harness the utility and multiple applications associated with smartphones. 

One limitation to studies of this nature is the potential difficulty in generalizing some of the findings. This has been highlighted by other authors in the past [31], emphasizing the small sample sizes of some of the studies and the limited population being studied [36, 37], although there have been robust findings in at least one study with greater power [26]. One particular concern is patient confidentiality, as their personal cell phone may not always be on their person, and access by other people to reminders on the mobile device may be a sensitive issue [25]. This is especially salient given the nature of the illness and the potentially revealing messages that may be sent to patients. On the other hand, some authors have found that the vast majority of patients do not feel that their privacy is being intruded upon [34]. Either way, there are many options which can be utilized to better protect patient privacy (a concern which has been raised before) [38]. Another limitation of the study is that we did not collect information on the total number of missed appointments an individual had up until the start of the study. This information may have improved our understanding of adherence behaviors. Finally, we did not collect information regarding HIV risk factors or biological markers (i.e., viral load or CD4 count) of disease. 

## 5. Conclusions

Future directions include incorporation of ever-evolving technology as it becomes increasingly accessible to the general population. PLWH have shown they are open to employ these strategies in the management of their illness [34]. Development of interactive, economically viable options for PLWH has been shown to be a very promising field, which could benefit a significant portion of the HIV/AIDS population. The vast majority of patients in an urban HIV clinic own mobile phones and would use them to enhance adherence interventions to HIV medication. In addition, our study indicates that smartphone use is becoming a reality for this population, which could open new avenues for technology-based interventions, though continued data collection through well-organized RCTs is needed.

## Figures and Tables

**Table 1:** Demographic characteristics of study sample.

Demographic characteristic	
Age (mean ± SD)	46.4 ± 9.2
	
Gender (*n* = 99)	
Male	62.6%
Race (*n* = 99)	
Black	93.0%
White	4.0%
Other	3.0%
Ethnicity (*n* = 79)	
Hispanic	11.4%
Education (*n* = 99)	
<High school	36.3%
High school/GED	37.4%
>High school	26.3%
Income ($/year) (*n* = 99)	
<10,000	67.3%
10,000–30,000	23.5%
>30,000	9.2%
Taking HAART (*n* = 99)	83.5%
Current smoker (*n* = 98)	57%

**Table 2:** Reasons given for using their cell phone.

Reasons (*n* = 96)	%
Call out or receive phone calls	92
Camera or video camera	64
Send or receive text messages	59
Clock	49
Calendar/scheduling	48
Alarms	47
Calculator	41
Access the Internet	34
Listen to music	32
Send or receive email	29

**Table 3:** Response to the AACTG Adherence Instrument among participants who reported ever missing a dose of HIV medication (expressed in percentages).

List of reasons (*n* = 25)	Never	Rarely	Sometimes	Often
Simply forgot	8	40	52	0
Fell asleep/slept through dosage	16	32	52	0
Away from home (*n* = 23)	30	22	39	9
Had a change in daily routine	36	20	40	4
Fell ill or sick	42	23	31	4
Felt depressed/overwhelmed	52	12	28	8
Did not want others to notice me taking pills	68	8	12	12
Had too many pills to take	68	8	24	0
Wanted to avoid side effects	64	16	20	0
Had problems taking meds at specific time	48	32	20	0
Felt drug was toxic or harmful	76	12	12	0
